# Meaning in Life for Patients With Severe Dementia: A Qualitative Study of Healthcare Professionals' Interpretations

**DOI:** 10.3389/fpsyg.2021.701353

**Published:** 2021-09-01

**Authors:** Tor-Arne Isene, Sigrid Helene Kjørven Haug, Hans Stifoss-Hanssen, Lars J. Danbolt, Liv S. Ødbehr, Hilde Thygesen

**Affiliations:** ^1^Centre for Psychology of Religion, Innlandet Hospital Trust, Hamar, Norway; ^2^Centre of Diakonia and Professional Practise, VID Specialised University, Oslo, Norway; ^3^Faculty of Social and Health Sciences, Department of Health and Nursing Sciences, Inland Norway University of Applied Sciences, Elverum, Norway; ^4^MF Norwegian School of Theology, Oslo, Norway; ^5^Faculty of Health Sciences, Department of Occupational Therapy, Prosthetics and Orthotics, Oslo Metropolitan University, Oslo, Norway; ^6^Faculty of Health Studies, VID Specialised University, Oslo, Norway

**Keywords:** severe dementia, person-centred care, residential care, meaning in life, meaning-making, meaningfulness, personhood

## Abstract

The need for meaning in life is a key aspect of being human, and a central issue in the psychology of religion. Understanding experience of meaning for persons with severe dementia is challenging due to the impairments associated with the illness. Despite these challenges, this article argues that meaning in life is as important for a person with severe dementia as it is for everyone else. This study was conducted in a Norwegian hospital and nursing home context and was part of a research project on meaning in life for persons with severe dementia. The study builds on two other studies which focused on how meaning-making and experience of meaningfulness appeared in patients with severe dementia. By presenting the findings from these two studies for a group of healthcare professionals and introducing them to research on meaning in life, the aim of this study was to explore how healthcare professionals interpret the patients' experience of meaning in life in practise for patients with severe dementia in a hospital and nursing home context, and to highlight its clinical implications. The study was conducted using a qualitative method with exploratory design. The data were collected at a round table conference, a method inspired by a mode of action research called “co-operative inquiry.” Altogether 27 professional healthcarers, from a variety of professions, with high competence in dementia care participated together with six researchers authoring this article. This study revealed that healthcare professionals were constantly dealing with different forms of meaning in their everyday care for people with dementia. The findings also showed clear connexions between understanding of meaning and fundamental aspects of good dementia care. Meaning corresponded well with the principles of person-centred care, and this compatibility allowed the healthcare professionals to associate meaning in life as a perspective into their work without having much prior knowledge or being familiar with the use of this perspective. The study points out that awareness of meaning in life as an integrated perspective in clinical practise will contribute to a broader and enhanced repertoire, and hence to improved dementia care. Facilitating experience of meaning calls for increased resources in personnel and competence in future dementia care.

## Introduction

Today there are about 50 million people worldwide living with dementia and the number is projected to increase to about 152 million by the year 2050 (Patterson, 2018). In Norway, which is the context of the present study, almost every seventh person (14.6%) aged more than 70 years lives with dementia, and for the age group 90 years and older the prevalence increases to one out of two persons (48.1%) (Demensplan 2025 [National plan for dementia care 2025], 2020). In nursing homes five out of six residents (84.3%) are living with dementia (Demensplan 2025 [National plan for dementia care 2025], 2020). Seeing dementia as an increasing threat to the public health the Norwegian government aims to “*create a society that to a greater extent promotes mental and physical health by facilitating coping, belonging and experience of meaning*” [Authors' translation^1^] (Demensplan 2020 [National plan for dementia care 2020], 2015, p. 9). Seeing experience of meaning as a contributor to health, it is important to investigate how this experience can be facilitated into the care of persons with severe dementia.

The need for meaning in life is a key aspect in being human, and a central issue in the psychology of religion (Park, 2005). Victor Frankl described *will to meaning* as the “*man's primary concern”* (Frankl, 1969, p. 20). Understanding experience of meaning for persons with severe dementia is challenging due to the impairments associated with the illness. The severity of symptoms of dementia is classified in stages from mild, moderate to severe dementia (WHO, 1993 in Hughes et al., 1982; Engedal and Haugen, 2018). For persons with severe dementia the capacity for cognitive functioning and abstract thinking is severely impaired, and the ability to verbally communicate or express oneself is reduced (Kitwood and Bredin, 1992; Engedal and Haugen, 2018). Despite these challenges, seeing meaning in life as a key aspect in being human, this article argues that meaning in life is as important for a person with severe dementia as it is for everyone else. Healthcare professionals' awareness and knowledge about meaning in life brings an important perspective into person-centred care for this patient group. This study is not patient-oriented but seeks to bring out knowledge based on healthcare professionals' experiences and understanding of the topic.

## Background

The traditional thinking about dementia has been dominated by a focus on individual neuropathology and resulted in overlooking the important role of external factors on disease progression and loss of self (Kitwood, 1997; Kontos, 2005). Over the last decades these attitudes have changed towards seeing persons with dementia from a social perspective, affirming the personhood and identity even while cognitive functions are lost (MacKinlay and Trevitt, 2012). Dementia has social implications as it can affect how society treats people with dementia, and thus making it a relational disability (Swinton, 2011). Seeing dementia as a relational and social illness, Kitwood (1997) has influenced the changes towards a perspective of “person-centred care,” based on meeting the main psychological needs of love, comfort, identity, occupation, inclusion, and attachment. Person-centred care is an approach that sets the persons at the centre where the individuals are supported, facilitated, and enabled to contribute to their own care (Mitchell and Agnelli, 2015). Dementia care should, according to Kontos, be this kind of “person work” (Kontos, 2005), with an ultimate goal of “offsetting the fragmentation of selfhood” (Kitwood and Bredin, 1992). In Norway, the principles of person-centred care form the basis of policy on dementia care. While meaning *of* life is a philosophical question and not possible to prove by any methods, empirical research deals with meaning *in* life (Schnell, 2021). Since Frankl (1969), different aspects of meaning in life have been investigated by a number of researchers (Crumbaugh, 1968; Battista and Almond, 1973; Antonovsky, 1993; Schnell, 2009, 2021). Meaning in life in relation to different health aspects is associated with a variety of physical and psychological outcomes (Steger, 2012; Czekierda et al., 2017). Scandinavian research on meaning in life has been linked with existential issues in different clinical populations (DeMarinis, 2008; Haug et al., 2016; Lloyd, 2018). Recent studies that included cognitively intact nursing home residents have shown that perceived meaning in life is significantly associated with nurse-patient interaction (Haugan, 2014b; Haugan et al., 2020).

Research on meaning and dementia tends to focus on meaning for the caregivers or the caregiving process (Butcher and Buckwalter, 2002; McLennon et al., 2011; Butcher, 2016; Cherry et al., 2019). However, some research on meaning for the person with dementia has come up the last few years, showing that meaning in life provides life satisfaction and reduces depressive symptoms for persons with Alzheimer's (Dewitte et al., 2019). Research has also pointed out that relationship and connectedness are of great significance for the experience of meaning for persons with dementia (MacKinlay, 2016). A study on how the view of life frames the sense of identity of persons with Alzheimer's in telling their life-story, showed an experience of meaningfulness and continuity when looking back on their lives (Westius et al., 2010). Several studies on meaning also refers to spirituality or spiritual needs, as these are closely related (Kevern, 2015). In the present study spirituality is not highlighted but is seen as a subordinate concept as a mean or source of meaning (Schnell, 2009).

Over the last 15 years Schnell and her collaborators have done comprehensive research on meaning in life (Schnell and Becker, 2007; Schnell, 2009, 2021). The concept of meaning in life is understood as a multidimensional construct consisting of the following dimensions: “*Meaningfulnes*s,” “*Crisis of Meaning*,” and “*Sources of Meaning”* (Schnell, 2021, p. 6–8). Early empirical studies on meaning in life assumed that meaningfulness and crisis of meaning were opposite sides of the same continuum (Schnell, 2009, 2021). Schnell's research has found the two dimensions to be relatively independent as low meaningfulness and low crisis of meaning may occur together (Schnell, 2009, 2021). Meaningfulness is based on an “*evaluation of one's life as coherent, significant, oriented and belonging*” (Schnell, 2009, p. 487; Schnell, 2021). These four criteria can be understood as central elements to the experience of meaningfulness without referring to its sources (Schnell, 2021). A crisis of meaning is defined as “*a judgement of one's life as frustratingly empty, pointless and lacking meaning*” (Schnell, 2009, p. 487; Schnell, 2021, p. 8). Schnell has identified 28 sources of meaning which are understood as orientations that correlates positively with meaningfulness and give form to peoples' actions, convictions, and experiences (Schnell and Becker, 2007; Schnell, 2021). The sources of meaning that are strongest associated with meaningfulness are generativity, care, religiosity, and harmony (Schnell, 2021).

Meaning-making is also a central concept in research on meaning (la Cour and Hvidt, 2010; DeMarinis, 2013). In this study meaning-making refers to meaning both as the experience of meaning in life and the process of creating it. Meaning in life is always experienced within a context and depends on the accessibility to sources of meaning. Being a resident in a nursing home or a patient in hospital may affect the access to sources of meaning as the residents are distanced to their normal living environment and are thus in an alienating situation. Living with dementia may also be experienced as a kind of alienation and affect the person's existence at a deeper level (Svanström et al., 2013). This is in particular the case for persons living with severe dementia, due to the severity of the cognitive impairments that characterise this phase. In relation to dementia, Schnell's understanding of meaning in life is useful because it emphasises the experience of meaning without necessarily having a cognitive awareness of why or what it is that creates meaning.

With exception of the few examples mentioned above, research on meaning in life and dementia is scarce in general, and particularly in relation to severe dementia. Some research has focused on how nurses understand residents with dementia's needs for meaning (Ødbehr et al., 2014). Ødbehr found that although the residents' needs for meaning were similar to the general population, retrieving and creating sources of meaning may be limited by dementia (Ødbehr et al., 2014). Empirical knowledge on how these needs for meaning in life are managed in dementia care practise is lacking. Exploring this knowledge gap, this study was part of a research project on meaning in life for persons with severe dementia. It is not possible to have direct knowledge about the patients' own experiences of meaning in life. However, exploring qualified healthcare professionals' perspectives provides the best knowledge accessible, and we find it important to bring this knowledge into the field of dementia care. The aim of this study was to explore how healthcare professionals interpret the patients' experiences of meaning in life in practise for patients with severe dementia in a hospital and nursing home context, and to highlight its clinical implications.

## Materials and Methods

The present study builds on two other studies within the same research project, all with a focus on how meaning-making and experience of meaningfulness appeared in patients with severe dementia. The two previous studies involved participant observation of patients and interviews with healthcare professionals at a hospital ward, with particular focus on how meaning-making and experiences of meaningfulness appeared in patients. Unlike the two other studies that collected data from observations of patients with severe dementia, the empirical data in this present study were based on healthcare professionals' understandings and interpretations of meaning in life for this patient group.

### Study Design

This study used a qualitative method with an exploratory design as it was found appropriate when investigating healthcare professionals' understandings and interpretations in a field where research is relatively scarce. A round table conference was held as this is a method which has proved to be relevant and fruitful for knowledge development in healthcare practise (Hummelvoll, 2006). The round table conference was carried out in one day and organised in three sections:

- Teaching with presentation of the project and introduction to the topic of the research (90 min).
- Discussion in three simultaneous focus groups (90 min).
- Plenary discussion (45 min).

Round table conference is a method inspired by a mode of action research called *co-operative inquiry* (Hummelvoll, 2006). Bringing in research relevant in a clinical setting, clinicians are invited to contribute with their professional experiences and together with researchers discuss and reflect upon how theoretical perspectives are relevant in practise. Focus groups are useful as research method when exploring issues concerning common experiences and perspectives in an environment where different people or professions cooperate (Malterud, 2012a). The purpose of the round table conference was to explore how meaning-making and meaningfulness in persons with severe dementia are relevant in dementia care, based on findings from the two earlier studies in this project.

### Collection

The data for this study were collected at a round table conference with healthcare professionals from a specialised dementia ward at an old age psychiatric department in Innlandet Hospital Trust, and healthcare professionals from the SAM-AKS network^2^. Altogether 27 healthcare professionals participated together with the six researchers authoring this article. The participants were nurses, occupational therapists, social educators, and psychiatrists working with persons with dementia. All age groups from early thirties to late sixties were represented, with some of the participants having more than 30 years of experience in dementia care. Using the SAM-AKS network, this sample included participants with high formal and clinical competence in dementia care from a variety of professions covering both the primary healthcare and specialist healthcare service.

Although different professions were represented among the participants of the focus groups, the moderators did not experience any power imbalance (which could be typical between doctors and nurses). The healthcare contexts from which the participants were recruited practised a relatively flat structure, as is normal in a broad part of the Norwegian healthcare system. The focus group interviews were conducted on neutral ground and the participants wore their private clothes. As all participants were given the chance to talk in rounds, the moderators felt that no particular profession notably dominated the conversation.

Findings from two other studies in this project on meaning in persons with severe dementia were presented at the round table conference. There were given examples of meaning-making and experiences of meaningfulness found in everyday situations with persons with dementia. Two introductions on *Worldview and health* and on *Meaning in life* were also presented. The participants were primed with Schnell's theory on *Sources of meaning* and understanding of *meaningfulness* (Schnell, 2009, 2011). The participants were also presented with the thinking around worldview and how it relates to and affects people's health (Stifoss-Hanssen and Kallenberg, 1998). The purpose of the teaching with presentation and introductions was to give a context and build an understanding of the content of the research question.

Following the teaching, the participants were interviewed in three different focus groups. Each group had 9 participants with a mix of professions and were moderated by two researchers from this study. The focus groups first shared their own experiences and stories of meaning-making in persons with dementia. They were then asked about what it is in these stories that makes us understand them as meaningful. Finally, the focus groups were asked how worldview and existential themes can be managed in persons with severe dementia, based on the findings they were presented for ahead of the focus group interviews.

Three main points from each focus group were presented in a plenary session, followed by an open discussion that acknowledged and elaborated the discussions from the focus groups.

### Analysis

Audio recordings from the focus group interviews and the plenary discussion were transcribed into 80 pages of text. The transcribed texts were analysed using *Systematic text condensation (STC)*, which is a pragmatic method of thematic cross-sectional analysis of qualitative data described by Malterud (2012b, 2017). The analysis was done in four steps: (1) Forming a total impression, (2) identifying and sorting meaning units, (3) abstracting the content from the individual meaning units, and (4) summarising the meaning. All the authors read through the material independently to form a general impression of the whole and identify preliminary themes. The authors then met to negotiate the meaning units and code groups. With an inductive and open-ended approach (Malterud, 2016), we looked for patterns and categories related to the concepts of meaning-making and meaningfulness, and the clinical implications of how the professionals understood and operated according to these concepts. The first author analysed the material by further sorting and abstracting the content from the individual meaning units, and together with the co-authors the meaning from the abstracts were summarised. The analysis was supported by the theories of meaning in life as developed by Schnell (2009, 2021).

The participants in this study were healthcare professionals working at hospital or nursing homes where the persons with dementia were referred to as *patients*. At the nursing homes *resident* was more common, but even here *patient* was frequently used. In this article we prefer to use *person with dementia* to validate humanity and the patient as an equal person. However, referring to situations and examples from the data material, we some places use the word *patient* to keep close to the material from an institutional context with asymmetrical relationships between healthcare professionals and the ones cared for.

### Ethical Considerations

Consistent with Norwegian legislation, the project was registered (#47488) and approved by the Norwegian Social Science Data Services (NSD) in April 2016. The project was also in 2020 registered by the Data Protection Office at Innlandet Hospital Trust. Participation was voluntary, and written informed consent was obtained from all participants before the data collection. The data were anonymised when transcribing the audio recordings into text.

## Results

The understanding of meaning and clinical implications of this understanding were identified through the analysis of the empirical material and organised in three main categories: “being on a treasure hunt,” “catching the moment” and “taking leadership.” The categories describe the approaches healthcare professionals used to search for what could be interpreted as meaning for patients with severe dementia. Again, it is important to emphasise that the findings are based on the participants' interpretations of the patients' experiences, as we do not have direct access the patients' own expressions. The presentation of results will be based on these categories. Although the categories will be presented separately, they may also overlap in practise.

### Being on a Treasure Hunt

The participants of the study shared an understanding that professional attentiveness for the patients' experiences and expressions of meaning was an important aspect of everyday dementia care practises. This attentiveness was described as an ongoing, but also uncertain process of searching for meaning, which involved a number of components.

The search for meaning meant that healthcare professionals interpreted and translated non-verbal—and often non-coherent verbal expressions—into something that could be understood as expressions of meaning for the patients. One of the participants described this ongoing work of searching for meaning as “*being on a treasure hunt*.” In this context using the analogy of being on a treasure hunt was understood as interpreting behaviour and expressions and catching seemingly trivial incidents as something potentially meaningful:

“*But you see, the job you are doing there, the small seed you manage to either spread further or get hold on, it makes a big difference*.”

One other participant described the search for meaning as “*finding the key*” that could change the situation for the patient. Other participants had a similar understanding of searching for meaning as taking the time and effort to go beyond what was up in the open:

“*… we also take it a little further. (…) We are trained to be able to describe behaviour and see symptoms. But then, you must also have time to try to understand the meaning behind what happens. If you have the time for that, and the ability for it, you will catch something completely different.”*

The work of searching for patients' experience of meaning required both time and knowledge as well as patience. The participants used their formal training and understanding of dementia and the principles of person-centred care in the search for understanding patients' experiences of meaningfulness. They underlined how this meant that they continually worked to sort out expressions of meaning from the pathological and seemingly meaningless and chaotic, and to focus on catching on to the individual behind the illness.

The participants of the focus groups gave many examples of how they actively searched for and initiated something that could give the patients experiences of meaning through everyday activities such as working in the garden, going for walks, celebrating birthdays, using humour and laugh of situations, singing or listening to music. Some activities, such as knitting and needlework, were experienced as best fitted for women. For men, they often chose more physically demanding activities like carrying laundry or garbage or sweeping the floor.

The participants also expressed how they searched to facilitate meaningful experiences for the patients by mobilising an important patient resource: personal background information about each of the patients, including their interests and previous occupation, as well as family background. This information provided the participants with clues as to where to look for meaning, and a context for interpreting potential meaning-making episodes.

The participants shared many stories describing how they succeeded in finding meaning. However, the participants pointed out that their caring-time was also spent being present in chaotic situations without finding meaning, as described in this quote:

“*There are also many times that one does not reach the goal, in searching for meaning, that the expression simply becomes too chaotic to put it into perspective*.”

They emphasised that the meaningful treasure-moments were fugacious and represented only tiny parts of the caring process, and that they had to endure the patients' chaos and meaninglessness between these moments. The participants also pointed out that a part of enduring the chaos of dementia care was about understanding and containing the patients' painful expressions, in order to ease or relieve the pain. Expressions of pain was understood as a way to communicate when other means of verbal communication were impaired. Participants faced the pain of their patients through a two-sided attention, where one aspect related to trying to reveal a possible underlying bodily cause, and the other aspect had to do with having an awareness that it may be an expression of inner or emotional pain.

### Catching the Moment

The second main category was that the process of meaning-making was understood in relation to what we have named “catching the moment.” The importance of the “here and now” or “there and then” and of being able to catch these moments of meaning was a recurring theme as illustrated by one participant:

“*… what is special about people with advanced dementia is that the meaning is there and then. The experience is there and then (…) So I think you have to look at the moment there and then.”*

We found that the participants related to catching the moments in three ways: *Enlarging the moments, seeing the moments as whole stories*, and *creating the moments*. The understanding of catching the moment involved more than the participants being aware of—and making use of—here and now moments. For example, the participants worked to *enlarge* the meaningful moments for the patients by making the most out of trivial occasions. One participant expressed the importance of everyday situations this way:

“*My experience is that it doesn't have to be that much hocus pocus, but everyday perfectly adequate things. Whether it's birthday celebrations or whether it's working in the garden, telling a joke, having something to laugh at. [Then] such everyday things that you take for granted in everyday life, is what creates meaning for everyone.”*

Unlike the patients with dementia where it was the here and now that counted, the participants had the possibility of a broader perspective; *seeing the moments as whole stories*. The participants considered the moments as tiny bits of a bigger picture of meaning, where it was possible to see patterns and resources to build on. The importance of knowing the background and life-story of the patients was a common feature. This was described as *deep diving* or *signing into* the patient's life-story. By signing or inviting oneself into the life-story of the person with dementia, the participants observed that there was meaning in the patients' activity. This made it possible to understand their behaviour, and thus to participate in creating and confirming a coherence for the patients. One of the participants illustrated this point by telling a story about one of the patients who spent much time crawling on the ward floor at the hospital. The healthcare workers felt sorry for the man and were worried about what his family would say if they found him crawling on the floor. But when they tried to help the man onto his feet, he became very distressed. However, the crawling on the floor all gave more sense when his family told them that the man had been a professional floor layer in his working years. While crawling on the floor he was actually at work. In this case, catching the moment was not sufficient to understand the meaning there and then. However, the patient's background provided a context for meaning that was not evident out of the situation but had to be searched for. Knowing the patient's background made it possible to interpret the crawling on the floor as a meaningful moment. The story showed that it is important to not only consider the moments isolated but seeing them as a part of a bigger picture. Seeing the moments as pieces of a whole story was also seen to give a sense of orientation as described by one of the participants:

“*But I think that maybe we easily think that setting direction and goals, we can't do that. But we are doing it all the time because it's in small pieces.”*

In addition to enlarging potentially meaningful moments for the patients in everyday situations, the participants also worked on *creating these moments*. The participants used different milieu-therapy tools, such as reminiscence activities or music, to achieve meaningful moments. Creating moments is related to the former category of being on a treasure hunt where everyday activities were used in search of meaning. As the everyday activities were initiated by the participants, one could also say that they were created moments.

### Taking Leadership

The third main category relates to the assignment of roles and responsibilities between participants and patients. The participants described the relationship between themselves and the patients as asymmetrical and they emphasised the need for taking leadership. However, the participants made nuanced descriptions of the components of this leadership.

Some of the participants described their role as being a “guide.” This role was seen as encompassing a number of responsibilities; including getting a grasp of the here and now situation of the person, simplifying or adapting choices in order to make them manageable for the person with dementia, offering opportunities for relief of pain and unease, as well as incorporating differentness and odd behaviours. In this way, the participants saw themselves as someone the patients could “lean on,” providing safety and meaning.

Taking leadership involved a demanding balancing act related to the participants' preservation of the personal integrity of the patient as described below:

“*It becomes such a delicate balance of taking leadership, but at the same time exercising or showing respect. They are persons who need management very much and guidance from the caregiver.”*

The participants identified certain characteristics of taking leadership. This was related to the participant's presence; of being attentive and physically present in order to be able to interpret non-verbal signals and behaviour. Some participants expressed the importance of “silent presence,” emphasising that being physically close to someone had a value in itself, without having to talk or do anything. By being present in silence the participants were taking responsibility for the relation to the patients through their presence.

The participants also described that taking leadership was about *tuning in*. “Tuning in” was characterised as the responsibility to “tune in on” the reality and situation of the person with dementia. However, the term was also used in reference to the task of preserving the demanding balance between leadership and personal integrity and dignity of the person with dementia. One of the participants used the word *resonance* as an expression of two-way communication based on observation of how the communication is received in the patient.

A common feature among the participants was to describe the relations between the participants and the patients as asymmetrical. This was partly done in reference to their role and responsibility as healthcare professionals. However, at the same time the participants expressed that the relationship between healthcare professionals and patients had changed compared to the past in terms of talking about the patients as *equal collaborators*. This seemingly contradictory understanding of balancing responsibility with collaboration was expressed in the following quote:

“*We have to regard them as equal partners, and we are the ones who are going to make them equal.”*

Here, in this context the notion of equal collaborators was used primarily as a stance towards recognising the identity and humanity of the patients, and in this way to the principles of human rights, emphasising the equal worth of all humans.

## Discussion

This study revealed that healthcare professionals were constantly dealing with different forms of meaning in their everyday care for patients with dementia. This finding corresponds to other research in the field (Haugan, 2014a,b; Ødbehr et al., 2014, 2015). We found that the understandings of meaning had clear connections with fundamental aspects of good dementia care, and that meaning was strongly associated with the principles of person-centred care. The participants were not familiar with seeing dementia care from the perspective of meaning-making and experiences of meaning. However, as the participants were primed on the subject in advance, their discussions showed that even if their work with person-centred care had not been identified as meaning, we found they dealt with central facets of meaningfulness, such as coherence, significance, orientation, and belonging (Schnell, 2009, 2021).

The aim of this study was to explore how healthcare professionals interpret the patients' experiences of meaning in life in practise for patients with severe dementia in a hospital and nursing home context, and to highlight its clinical implications. The findings of the study are important because they show how meaning in life can be facilitated into dementia care, and hence accommodate the Norwegian government's aim of facilitating experience of meaning as one of the measures to promote mental and physical health in society (Demensplan 2020 [National plan for dementia care 2020], 2015). The global prevalence of persons with dementia is projected to triple over the three next decades (Patterson, 2018). Based on the findings of this study it is our assumption that professional attentiveness for the patients' expressions and experiences of meaning contributes to improved dementia care. In the following we will discuss how meaning is facilitated in practise into the care of persons with severe dementia, as well as some implications for future dementia care.

### Attitudes of Openness and Respect

The way a healthcare professional sees a patient with dementia, will influence how the healthcare worker relates to this person and thus how the care is provided (MacKinlay and Trevitt, 2012). This is especially important when meeting people with severe dementia where the ability for verbal communication is limited and must largely be based on the interpretation of non-verbal behaviour. The participants in this study demonstrated attitudes of *openness* and *respect* towards the patients. Being open towards a reality that could be perceived as different from what they expected and attempting to understand what could exist behind odd behaviours, they got in touch with something that seemed to give meaning for the patients. The findings showed that it was about seeing beyond the symptoms and diagnoses and was, for example, expressed as “finding the key” to something that could give change—and maybe meaning. This openness seemed to be based on respect. The etymological meaning of respect (*re*- “back” + *specere* “look at”) means to see again, to see once more. The participants' respect for the patients seemed to be about not underestimating them but look once more to confirm their significance as persons, and also about showing respect for the patients' autonomy through balancing between support and preservation of the personal integrity. In line with Schnell's (2009, 2021) characterisations, the results of openness and respect seemed to encompass aspects of meaningfulness. Openness towards looking beyond chaotic expressions and odd behaviours seemed to create contact with the identity and reality of the patients and thus produce a sense of *significance* and *coherence*.

### Participating Partnership

The study showed that the practise where the processes of meaning-making and experiences of meaning happened was a cooperative teamwork between the participants and the patients. Being on a treasure hunt, catching the moment, and taking leadership—the categories of findings are descriptions of how the participants were interpreting and managing meaning in their role and relations to the patients. Seeing themselves as guides for the patients to lean on, the participants highlighted qualities like openness, presence, equality, interaction, and generosity in relation to the patients' needs. Based on the attitudes of openness and respect the healthcare professionals were facilitating meaning through active participating in a partnership with the patients. This participating partnership built on the principles of person-centred care, seeing dementia as a social and relational illness (Kitwood, 1997). The findings of this study showed that the healthcare professionals were supporting the patients by taking leadership, being close, present, being able to endure chaotic situations, creating and enlarging moments, and keeping a broader picture of situations. In a person-centred perspective where this support may be interpreted as meeting psychological needs, we found that the healthcare professionals' contributions in this partnership were giving the patients experiences of meaning. This finding has been thoroughly tested and verified in recent studies by Haugan et al., which included cognitively intact residents in nursing homes and showed that perceived meaning in life is significantly associated with perceived nurse-patient interaction (Haugan, 2014b; Haugan et al., 2020).

This cooperate teamwork between the participants and the patients connects to the social dimention of meaning in life (Schnell, 2021). Meaning in life is not only explored, experienced, or expressed individually, but can be understood as a social interactive event. The ones caring for the persons with dementia, the context and physical environment, as well as artefacts, are all carriers of meaningfulness. This social dimention of meaning is perhaps particularly important for persons with cognitive impairments. According to Schnell, being recognised and held in an interpersonal relationship is important for a person's experience of meaning, likely by strengthening the sense of *belonging* and *significance* (Schnell, 2021).

Schnell (2021) refers to *belonging* as an existential experience more than the social aspect of it. In this study, the participating partnership seemed to hold both the social and the existential aspects of belonging as it provided the patient with the physical experience of being present together with someone, as well as the experience of having a place in the world and thus also being of *significance*. As the reality becomes more fragmented as a consequence of the progression of dementia, it challenges the senses of *coherence* and *orientation*. However, the interactions in the partnership with the healthcare workers seemed to hold the fragments together giving a sense of *coherence* by making use of the patients' background information and *orientation* by giving the moments purpose.

This study relied on the healthcare workers' understandings and interpretations. However, the focus in this participating partnership was, in line with the first part of the aim in this study, on the patients' experience of meaning in life and not the healthcare workers'. Although the participants found it rewarding to work with the patients on the issues of meaning, their role in the partnership was to facilitate these experiences for the patients.

### Significance of Meaning in Dementia

Meaning is created and experienced within a paradigm or context, which in this study was within a population living with severe dementia. Understanding meaningfulness in line with Schnell (2009, 2021) as experiences of *coherence, significance, orientation* and *belonging*, one could easily think that people with severe dementia are bereaved of these experiences. The findings of this study demonstrated that healthcare professionals considered that—even if meaning for persons with dementia in several ways is affected by the illness—it is nevertheless of significance and has the same functions as meaning has for other people. The participants of this study focused on sorting out expressions of meaning from the pathological. This often meant facing and containing chaotic and painful expressions. Expressions of pain in the patients were approached with a two-sided attention. On the one hand the healthcare professionals observed possible unmet needs or underlying bodily causes. On the other hand, it meant going beyond this perspective seeing it as expressions of inner and emotional tensions. This two-sided attention opened up for the possibility that the patients were experiencing a crisis of meaning.

Using the analogy of treasure hunt as one of the categories of findings, the uncertainty of the process of searching for meaning and finding potential sources of meaning was highlighted. The participants had no means of knowing what was valuable, but had to interpret, continually, the patient's behaviours and expressions. Using the background and life-story of the patients along with different milieu-therapy tools such as reminiscence activities seemed to give access to various sources of meaning that was no longer conscious to the patients.

Seeing meaning in life to be as important to persons with dementia as it is for other people highlights significant challenges in dementia care, this study revealed that giving patients with severe dementia experiences of meaning in life was not happening by itself but had to be initiated and guided by others. Being attentive and facilitating the perspective of meaning in life takes a lot of resources, time, and effort. Coming from both the specialist health service and the SAM-AKS network in the primary health service, the participants in this study possessed high levels of professional competence. The normal situation in the municipal primary healthcare may be quite different as this is a sector with a large proportion of unskilled labour, often working part time, and with a high turn-over of personnel. Accommodating the government's goal of facilitating experience of meaning into dementia care could prove to be difficult due to lack of resources by today's standards.

### Strengths and Limitations

Findings from this study cannot be generalised which is not the intention of qualitative investigations based on interpretations from healthcare professional with the purpose of gaining an understanding of the clinical implications of meaning in dementia care. This study was not patient-directed but aimed at getting new qualitative knowledge about the professionals' experiences, interpretations, and interventions. Nevertheless, the participants in this study represent a wide range of professions with high competence in dementia care which provided the best knowledge accessible from interdisciplinary perspectives. The study used a cross-sectional method of analysis and did not emphasise potential differences between professions. Although it could be interesting looking into interdisciplinary differences, we think that the strength of having perspectives from this variety of professions are the outcome of accumulated knowledge from this group. The choice of round table conference as a multi-step method with the possibility to discuss each other's contributions from the focus groups, in addition to the group of authors cooperating in all steps including data collection, analysis and writing process of the article, provided a high degree of reflexivity to the study. As the participants of the study provided interdisciplinary perspectives together with the research group cooperating in all levels from data collection to authoring this article, this study offered new insights that contributed to our understandings of meaning in life in dementia care. Priming the participants on theories of meaning in life and focusing the discussions within this concept provided knowledge with high specificity. A limitation caused by this specificity is that potential related knowledge was not captured. The study provided internal validity through a high degree of information power according to the criteria as proposed by Malterud: aim of the study, sample specificity, use of established theory, quality of dialogue, and analysis strategy (Malterud et al., 2016). The external validity of the study was met by strategical selection of sample in order to give qualified answers to the research questions.

## Conclusion

The study showed that healthcare professionals' interpretation of meaning in life in persons with severe dementia corresponds well with the principles of person-centred care. This compatibility with person-centred care allowed the healthcare professionals who participated in the study to associate meaning in life as a perspective into their work without having much prior knowledge or being familiar with the use of this perspective.

Persons with dementia have full human dignity, and it is therefore important to keep them in personhood in order to meet them as whole human beings. The study showed that a perspective of meaning in life offers a broadened understanding of personhood and identity in persons with dementia. Contributing to a broader and enhanced repertoire in person-centred care, an awareness of meaning in life as a perspective in care of persons with severe dementia can improve clinical practise in the future.

The study revealed that facilitating experience of meaning for patients in dementia care requires high levels of competence and resources. Together with a rapidly growing population of people with dementia in years to come, this calls for a high priority of increased resources in personnel and competence in future dementia care.

## Data Availability Statement

The raw data supporting the conclusions of this article will be made available by the authors, without undue reservation.

## Ethics Statement

The studies involving human participants were reviewed and approved by Norwegian Social Science Data Services (NSD) in April 2016 (#47488). Registered by the Data Protection Office at Innlandet Hospital Trust 2020. The patients/participants provided their written informed consent to participate in this study.

## Author Contributions

T-AI: conceptualisation (equal), methodology—data collection (equal), transcribing data, analysis (lead), writing—original draught preparation (lead), and writing—review and editing (equal). SH, HS-H, LD, LØ, and HT: conceptualisation (equal), methodology—data collection (equal), analysis (equal), and writing—review and editing (equal). All authors contributed to the article and approved the submitted version.

## Conflict of Interest

The authors declare that the research was conducted in the absence of any commercial or financial relationships that could be construed as a potential conflict of interest.

## Publisher's Note

All claims expressed in this article are solely those of the authors and do not necessarily represent those of their affiliated organizations, or those of the publisher, the editors and the reviewers. Any product that may be evaluated in this article, or claim that may be made by its manufacturer, is not guaranteed or endorsed by the publisher.

## Footnotes

^1^Original text: “*Regjeringen legger vekt på å skape et samfunn som i større grad fremmer psykisk og fysisk helse gjennom å legge til rette for mestring, tilhørighet og opplevelse av mening.”*

^2^SAM-AKS is a cooperating network in Norway with 33 participating municipalities and Innlandet Hospital Trust. The networks' primary aim is better medical examination and treatment of residents in nursing homes. Secondly the network is developing and evaluating a model for interaction between the specialist health service and the primary health service with a focus on following up residents in nursing homes.
